# Light wavelength modulates search behavior performance in zebrafish

**DOI:** 10.1038/s41598-024-67262-9

**Published:** 2024-07-17

**Authors:** Matthew R. Waalkes, Maegan Leathery, Madeline Peck, Allison Barr, Alexander Cunill, John Hageter, Eric J. Horstick

**Affiliations:** 1 Department of Biology Morgantown, West Virginia University, Morgantown, WV USA; 2 Department of Neuroscience Morgantown, West Virginia University, Morgantown, WV USA

**Keywords:** Wavelength, Zebrafish, Behavior, Search behavior, Visual, Sensory, Non-visual, Sensorimotor processing, Sensory processing, Visual system

## Abstract

Visual systems have evolved to discriminate between different wavelengths of light. The ability to perceive color, or specific light wavelengths, is important as color conveys crucial information about both biotic and abiotic features in the environment. Indeed, different wavelengths of light can drive distinct patterns of activity in the vertebrate brain, yet what remains incompletely understood is whether distinct wavelengths can invoke etiologically relevant behavioral changes. To address how specific wavelengths in the visible spectrum modulate behavioral performance, we use larval zebrafish and a stereotypic light-search behavior. Prior work has shown that the cessation of light triggers a transitional light-search behavior, which we use to interrogate wavelength-dependent behavioral modulation. Using 8 narrow spectrum light sources in the visible range, we demonstrate that all wavelengths induce motor parameters consistent with search behavior, yet the magnitude of search behavior is spectrum sensitive and the underlying motor parameters are modulated in distinct patterns across short, medium, and long wavelengths. However, our data also establishes that not all motor features of search are impacted by wavelength. To define how wavelength modulates search performance, we performed additional assays with alternative wavelengths, dual wavelengths, and variable intensity. Last, we also tested blind larvae to resolve which components of wavelength dependent behavioral changes potentially include signaling from non-retinal photoreception. These findings have important implications as organisms can be exposed to varying wavelengths in laboratory and natural settings and therefore impose unique behavioral outputs.

## Introduction

Vision is the primary means by which most organisms assess their natural environment. To exploit visual information requires light^1,2^. Given light’s ubiquity, animals have adapted complex visual systems and a diversity of specialized proteins, known as opsins, to sense and process light in the environment^3,4^. However, light in the natural environments is not homogenous and is composed of a spectrum of electromagnetic energy^5,6^. Indeed, the light-sensitive opsins in photoreceptors preferentially respond to distinct wavelengths within the visual spectrum^7–9^. Specific wavelength sensitivity is further complicated by more recent work that also demonstrates that non-visual opsins, or deep brain photoreceptors, are also expressed throughout the brain of many vertebrates^10–14^. These non-visual photoreceptors retain function, preferential wavelength sensitivity, and ability to impact behavior. Prior studies in diverse species, including humans, have even shown differential functional responses in the brain to light of varying wavelength^15–19^. However, what remains incompletely understood is how different wavelengths can impact behavioral performance.

To investigate how specific wavelengths of light impact behavioral performance, we used larval zebrafish. Light is critically important to larval zebrafish as they are becoming obligate predators as they wean off yolk nutrients, and predatory success requires visual input^20,21^. Since light-mediated vision is the main stimuli used by zebrafish to hunt and feed, zebrafish have developed a characteristic light-search behavior to find sources of light, which is initiated by the loss of environmental illumination^14^. Larval zebrafish search patterns are consistent with search pattern behaviors observed in other species^22–28^. Moreover, like in other search strategies, larval zebrafish light-search shows an initial local search pattern that transitions to an outward exploratory strategy^14^. Both of these phases exhibit distinct locomotor patterns, which the etiological and environmental context has been thoroughly discussed previously^14,29^. Our prior work has shown that local search is driven by the retina, whereas the transition to outward search is mediated by non-visual photoreceptors. Other zebrafish visuomotor behaviors have been shown to be wavelength-sensitive, such as the optomotor response and prey-capture^30,31^. However, these behaviors are driven by ongoing reaction to visual input^31–33^. Conversely, how different wavelengths regulate state dependent behavior is not well explored^34^. Therefore, larval zebrafish and light-search behavior provides a powerful system to determine how specific wavelengths impact performance of etiologically relevant, sustained state-dependent behavior.

We exposed larval zebrafish to diverse narrow spectrum LED light across the visible light spectrum. Our results show that wavelengths at short, medium, and long wavelengths, relatively, have consistent impacts on local search motor patterns. To understand how different wavelengths impact search behavior performance, we tested multiple parameters including chronic versus acute wavelength exposure, dual wavelength exposure, and the impact on outward search performance and the transitional period between these search strategies. Finally, we assessed whether wavelength-driven behavioral changes were affected by deep brain photoreceptors by testing genetically blind zebrafish. Overall, we demonstrate that specific light wavelengths can drive varied changes in light search behavior in larval zebrafish and that long wavelengths in particular show a consistent attenuation of search responsiveness that is not completely dependent on retinal input. These results are impactful as they show that lighting conditions can impact behavioral performance, which has implications for how animals respond in both natural and laboratory settings.

## Results

### Wavelength of light modulates local search behavior

**Figure 1:**
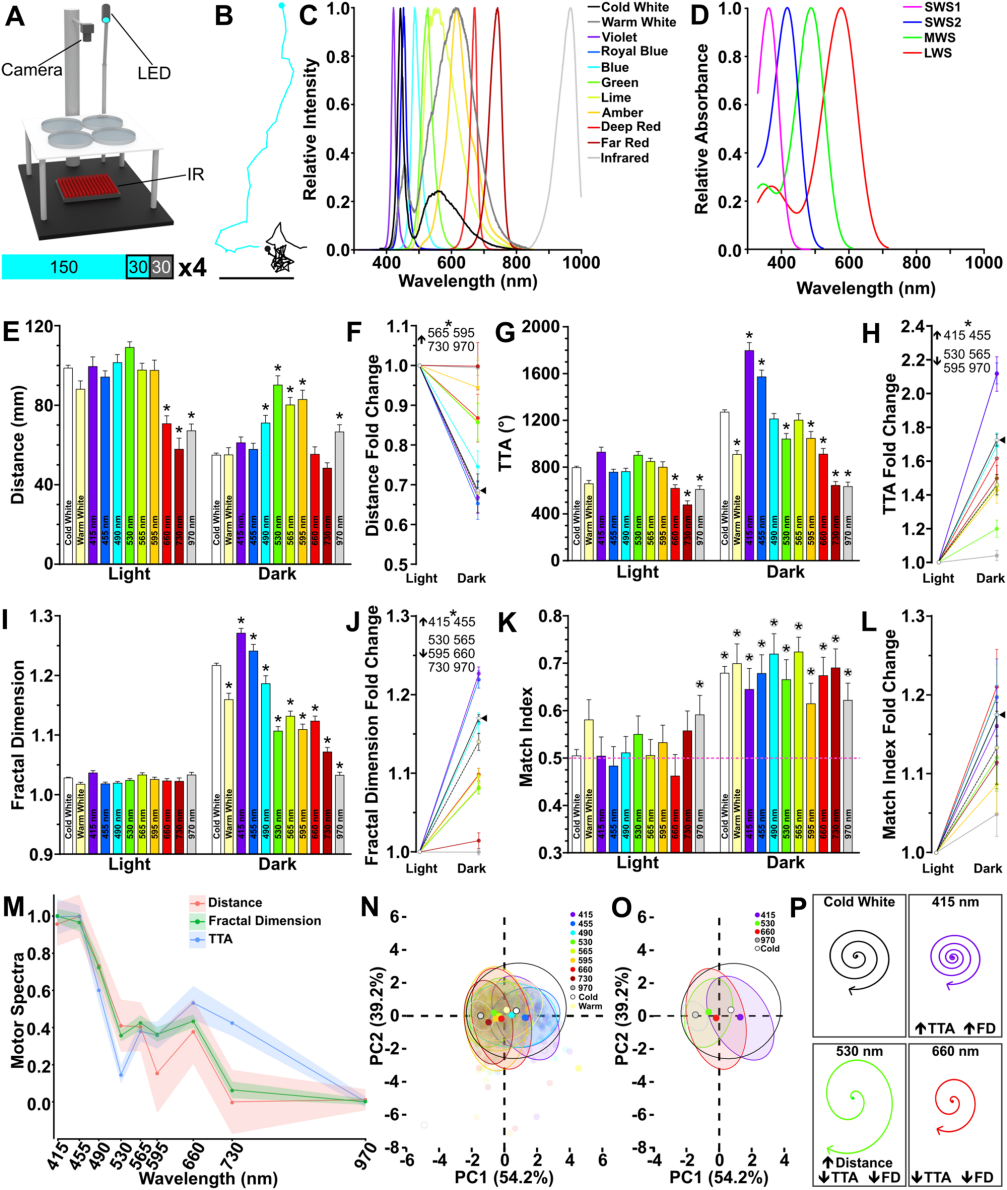
Light wavelength influences light search performance. (**A**) Behavior rig set-up shown with blue LED. Below: Light series used for recordings. Numbers shown are seconds. Blue signifies light ON periods while dark grey light OFF. Black outlines indicate active recording periods. Recording series was repeated 4 times (× 4) with a 150 s inter-trial interval with the respective light ON. (**B**) Example tracks seen in zebrafish during the light ON (blue) and after the loss of light (black). Scale bar 20 mm. (**C**) Emission spectra of 11 LED lights used for experiments. (**D**) Absorbance spectra of the four cone opsins in the larval zebrafish retina. (**E**–**J**) Quantification of distance (**E**, **F**), total turn angle (TTA) (**G**–**H**), and fractal dimension (**I**–**J**) during light and dark recording periods. Wavelength indicated on bars. Data is generated using the average across all four trials. (**E**, **G**, and **I**) show magnitude of different motor responses, whereas (**F**, **H**, **J**) show that change during the light to dark transition. Asterisk indicates *p* ≤ 0.05 compared to cold white light responses during baseline or dark periods. (**K**–**L**) Quantification of motor asymmetry magnitude (**K**) and transition (**L**). Circled asterisk indicates *p* ≤ 0.05 from 0.5 (random). Asterisk for all fold change figures show *p* ≤ 0.05 change that is greater than (up arrow) or less than (down arrow) cold white light controls. Individual wavelengths indicated next to arrow. (**M**) Motor spectra calculated using normalized fold change data for distance, fractal dimension, and TTA across all tested single wavelength lights. (**N**) Individual PCA plots for each wavelength based on fold change data for fractal dimension, distance, and TTA. Ellipses surround 90% of data points for each wavelength tested. Opaque colored circles represent the centroid plotted for each ellipse. (**O**) As in L, but subset 415 nm, 530 nm, 660 nm, 970 nm, and cold white light wavelengths. Percentages on axes represent variance of each principal component. (**P**) Schematic demonstrating how wavelengths selected for ongoing analysis impact local search performance. N’s: cold white light = 564, warm white light = 59, 415 nm = 64, 455 nm = 64, 490 nm = 68, 530 nm = 62, 565 nm = 77, 595 nm = 59, 660 nm = 59, 730 nm = 54, 970 nm = 65. In all figures error bars, or envelope in M, show standard error of the mean (SEM).

Motor patterns associated with search behavior are stereotypical across teleost species^28^. To assess the impact of wavelength on search motor patterns, we assay behavioral responses using 8 LEDs with narrow emission spectra spanning the visible range, along with a narrow infrared wavelength and broader spectrum cold white light and warm white light LEDs (Fig. 1A–C). This array of wavelengths provides comprehensive coverage of the zebrafish visual range, and also tests wavelengths that have variable partial overlap with the absorbance spectra of the visual opsins (Fig. 1D, Table 1). In our prior work we used cold white light^14,28,29,35,36^, which is used here as a control/standard light source. Individual larvae were tracked over four tandem light–dark intervals, their responses averaged per individual. The initiation of a local search response is characterized by decreased distance traveled, increased turning, and an increase in localized movement^14^. Therefore, we quantified distance, total turning angle (TTA), and fractal dimension (a measure of localized movement)^37^. We analyzed how wavelength impacted behavior during baseline illumination, dark responses, and light–dark transition strength. All tested wavelengths modulated motor outputs during local search compared to cold white light (Fig. 1E–J). As a negative control, we included 970 nm, a non-visible wavelength to zebrafish^33,38,39^. This control functionally recapitulates a constant dark environment. Exposure to this infrared wavelength produced no behavioral change across fictive ‘light’ and ‘dark’ recording intervals; indicating electromagnetic energy alone could not drive changes in search pattern motor outputs (Fig. 1E,J, Supplementary Table 1). Thus, the observed motor changes were tied to the sensation of each specific wavelength. Even the far red (730 nm) wavelength elicited changes in some, yet not all, motor metrics following light extinction (one sample t-test on TTA: *t*(53) = 6.63; *p* < 0.0001) (Fig. 1E–J, Supplementary Table 1). Therefore, 730 nm is likely near the limit of the zebrafish visually sensitive range. Together these data show that all tested wavelengths within the visible spectrum trigger local search behavior following light extinction yet have differential effects on motor performance during baseline and local search locomotion.

**Table 1:** Relative absorbance of tested wavelengths.

Line	Colur	λ (nm)	Relative abs at λ			
			SWS1	SWS2	MWS	LWS
	Cold white	N/A	N/A	N/A	N/A	N/A
	Warm white	N/A	N/A	N/A	N/A	N/A
	Violet	415	0.143	1.000	0.360	0.184
	Royal blue	455	0.002	0.444	0.819	0.166
	Blue	490	0	0.049	0.986	0.359
	Green	530	0	0.001	0.457	0.776
	Lime	565	0	0	0.078	1.000
	Amber	595	0	0	0.007	0.829
	Deep red	660	0	0	0	0.093
	Far red	730	0	0	0	0.001
	Infrared	970	0	0	0	0

Shows representative color, name, and peak wavelength of LEDs used in this study that correspond with Fig. 1C. For single wavelength light sources the relative absorbance at peak wavelength is shown for each visual cone opsin.

Another prominent feature of local search is persistent same-direction turning^14,29,36^. In our prior work, we have shown that an individual’s turn direction during local search is a stable motor preference or motor asymmetry. To examine if wavelength also affects the motor asymmetry, we used match index (MI) as in our previous studies^28,29,36^. For all visible wavelengths, motor asymmetry is present and comparable to cold white light controls in magnitude (Kruskal–Wallis *H*(10) = 9.50, *p* = 0.4858) and fold change between light and dark periods (Kruskal–Wallis *H*(10) = 11.36, *p* = 0.3300) (Fig. 1K–L). This data indicates that although wavelength does drive significant behaviorally changes, not all visuomotor responses are wavelength sensitive.

To further analyze the effect of wavelength on search performance we calculated a ‘motor spectra’ using the fold change data generated for TTA, distance, and fractal dimension for each of the single wavelengths tested (Fig. 1M). We found a significant main effect of wavelength (Two-way ANOVA—*F(8, 1689*) = 41.15, *p* < 0.0001), yet not motor metric (Two-way ANOVA—*F(2, 1689*) = 0.5902, *p* < 0.5902). The only exception was 730 nm light, which above we show is on the border of zebrafish visual sensitivity, where pair-wise comparison did show a significant difference between TTA and other measures (TTA vs. Distance adjusted *p* = 0.0083; TTA vs. fractal dimension adjusted *p* = 0.0313). When comparing experimental groups to cold white light, we qualitatively noticed several distinct behavioral patterns for short, medium, and long wavelengths. Short wavelengths (415–455 nm) drove motor patterns consistent with increased search activity (e.g., decreased distance travelled and an increase in TTA). Conversely, longer wavelengths (660–730 nm) produced patterns consistent with attenuated local search (Fig. 1E,G,I). Medium wavelengths (490–595 nm) also modulated local search parameters yet did not show uniform changes consistent with a general increase or decrease in search performance (Fig. 1E,G,I). For example, zebrafish exposed to 530 nm light had an increase in the distance travelled during the local search but a decrease in their turning and local movement. To test whether short, medium, and long wavelength responses potentially behaved as distinct groups we performed a Principal Component Analysis (PCA) using the fold change measures for all motor metrics, except MI (Fig. 1L, Supplementary Fig. 1). From this analysis, we observed that short and long wavelength clustered into relatively distinct groups, supporting our prior observations (Fig. 1N). Conversely, medium wavelength responses largely cluster with long wavelengths. In our analysis above, infrared had little impact on behavior, and in the PCA these responses formed a relatively distinct cluster from all other visible responses, suggesting the current PCA was capturing germane wavelength-dependent differences (grey circle, Fig. 1N). Based on the combination of PCA and observed changes in motor patterns we selected 415, 530, and 660 nm wavelengths for ongoing analysis as these wavelengths evenly covered the tested spectrum and represent the different behavioral patterns observed (Fig. 1O,P).

Previous work has suggested that short wavelengths are aversive to larval zebrafish^33,40,41^. Therefore, to ensure that our observations are not driven by differences in innate preferences between the wavelengths, we performed a phototaxis assay (Supplementary Fig. 2A,B). It is well-established that larval zebrafish show a strong positive phototaxis response to a focused spot of white light in a dark environment^42–44^. We found that each wavelength within our distilled testing group (415 nm, 530 nm, 660 nm, and cold white light) elicited positive phototaxis (Wilcoxon matched pairs signed rank test between time in ROI during baseline versus phototaxis conditions: white light *p* < 0.0001, 415 nm* p* < 0.0001, 530 nm *p* < 0.0001, 660 nm *p* < 0.0001) (Supplementary Fig. 2C). This data suggests that our focused set of wavelengths serve as a positive light stimulus to larval zebrafish, and therefore behavioral changes are due to wavelength-dependent modulation.

### Wavelengths acutely impact local search performance

**Figure 2:**
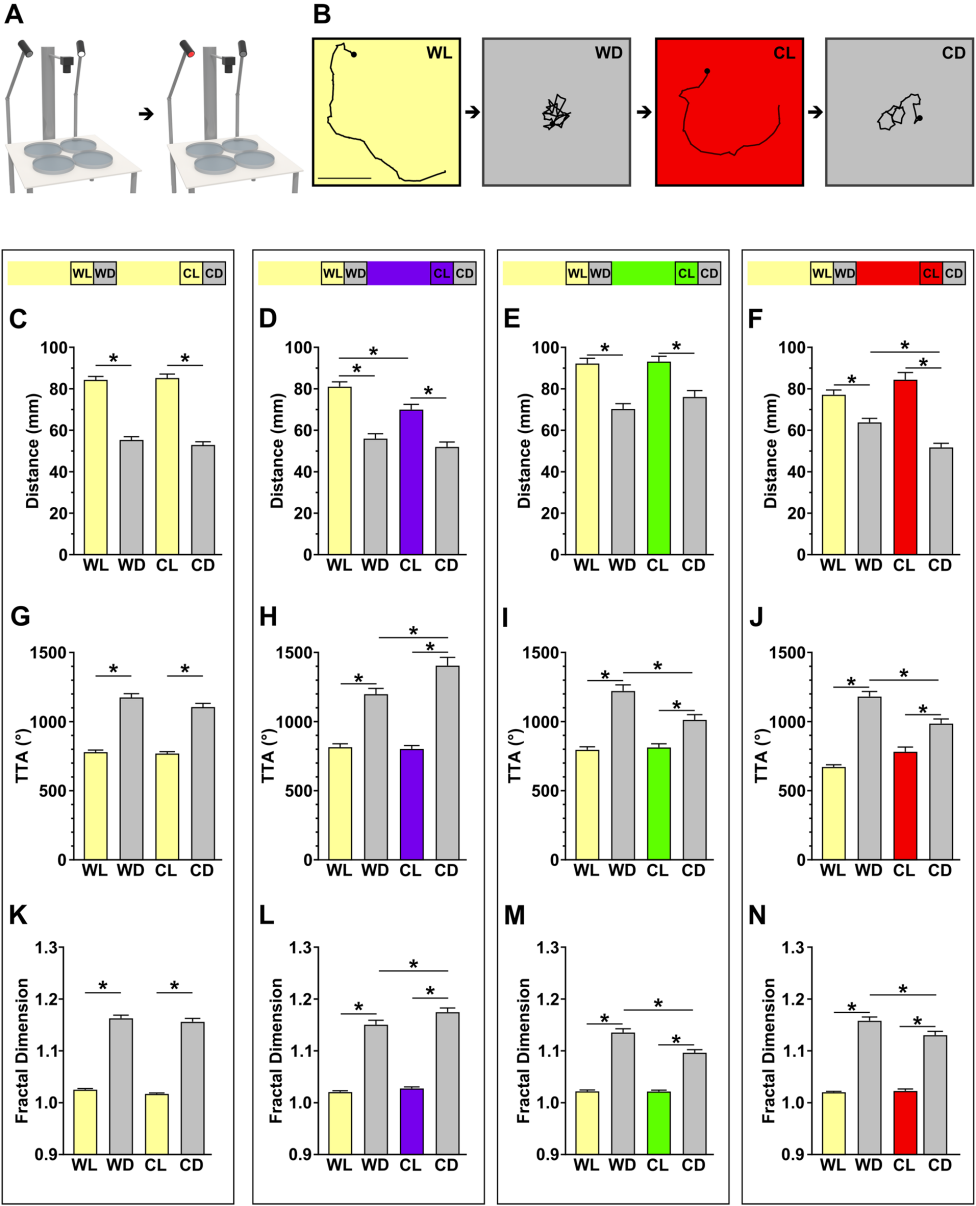
Distinct wavelengths can acutely modulate local search performance. (**A**) Behavior rig set-up with cold white and chromatic LEDs. (**B**) Representative traces for each recording period showing a red light example (WL: white light, WD: white to dark period, CL: color light, CD: color to dark period). Scale bar 20 mm. (**C**-**N**) Quantification of local search motor parameters across recording periods. (**C**–**F**) Distance, (**G**–**J**) TTA, and (**K**–**N**) fractal dimension showing white light, 415 nm, 530 nm, and 660 nm from left to right (color coded). The light series are above each column shows the lighting series and wavelength (color coded) for each experiment. Asterisks indicates *p* ≤ 0.05 between photoperiods designated. N’s: cold white light = 175, 415 nm = 94, 530 nm = 92, 660 nm = 102.

The changes seen in the local search behavior parameters thus far have occurred after an acclimation period of several minutes. To determine whether specific wavelengths can alter zebrafish search behavior outcomes in a more acute manner, we presented alternating cold white and colored light (Fig. 2A,B). This light sequence produces a series of white light (WL), white to dark (WD), color light (CL), and color to dark (CD) recording periods. In zebrafish, repeated presentation of stimuli can lead to habituation^45–48^. To determine how our extended testing may impact search motor parameters, white light controls were evaluated across the cumulative sixteen recording periods. Habituation, or a reduction in response strength, during baseline and local search was observed, yet significant changes stopped by the fourth trial for all metrics except distance (t-tests between fourth and eighth dark periods: distance *t(295)* = 3.791, *p* = 0.0002; TTA *t(295)* = 0.8191, *p* = 0.4134) (Supplementary Fig. 3). Therefore, habituation is likely to only have a minor impact during the extended light series.

From our acute testing paradigm, local search responses to changing light wavelength were largely replicated compared to our original tests (Fig. 2C–N). However, some deviations were observed, such as during 415 nm light ON intervals and 660 nm light OFF intervals where wavelength exposure caused reduced distance compared to paired white light (One Way ANOVA—415 nm: *F(3, 372)* = 31.86, *p* < 0.0001, WL vs. CL adjusted *p* = 0.0057; 660 nm: *F(3, 404)* = 35.00, *p* < 0.0001, WD vs. CD adjusted *p* = 0.0030) (Fig. 2D,F). This change could arise from certain wavelengths differentially impacting habituation over time. Alternatively, when comparing the white light period (WL) between groups, there is a significant decline in distance and TTA in the 660 nm group compared to cold white light, suggesting that the exposure to 660 nm light is attenuating the white baseline response of subsequent trials (One Way ANOVA *F(3,459)* = 10.48; distance: adjusted *p* = 0.0295, TTA: adjusted *p* < 0.0001) (Supplementary Fig. 4). Intermittent exposure to 415 nm and 530 nm also imposes mild motor changes during white light paired responses (WL and WD) (Supplementary Fig. 4). Therefore, some form of conditioning may be driving some minor changes in motor output. However, the overall observations largely confirm our earlier observations and establish that wavelength dependent changes to search performance can occur rapidly from acute exposure to distinct light wavelengths.

### Reduced search at long wavelengths is intensity independent

**Figure 3:**
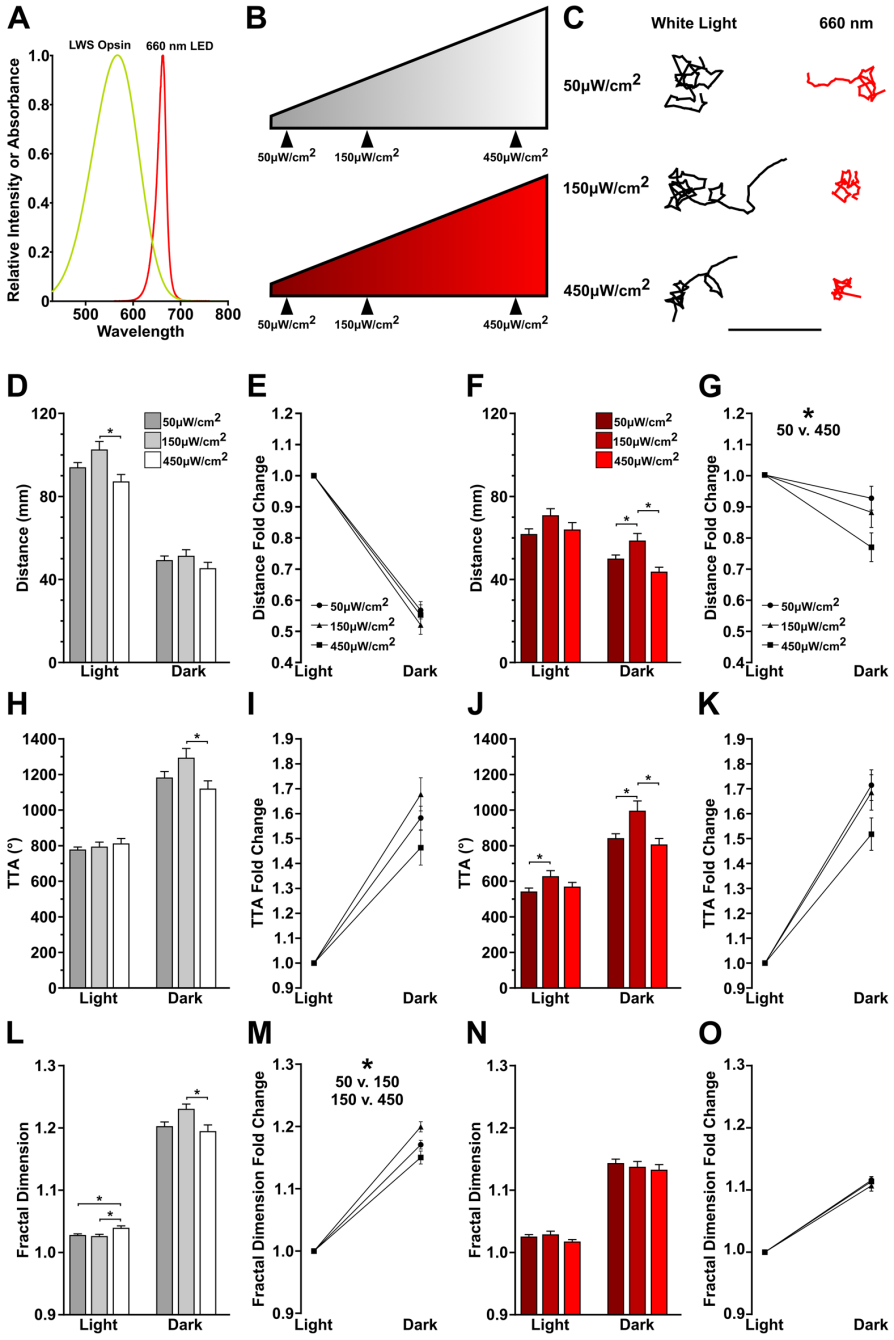
Long wavelength light modulates behavior independently of intensity. (**A**) Diagram of relative absorbance of the long wavelength stimulating (LWS) opsin and emission spectra of the 660 nm LED. (**B**) Schematic of tested cold white and 660 nm light intensities. (**C**) Representative traces of individual larvae following the loss of illumination. Traces show 30 s following the loss of light. Scale bar 20 mm. (**D**–**O**) Quantification of motor metric changes at varying light intensities. N’s: cold white light 50 µW/cm^2^ = 174, 150 µW/cm^2^ = 72, 450 µW/cm^2^ = 73; 660 nm at 50 µW/cm^2^ = 161, 150 µW/cm^2^ = 81, 450 µW/cm^2^ = 82. Asterisks indicates *p* ≤ 0.05 between designated intensities.

For an individual opsin, wavelengths across the full absorption spectra stimulate phototransduction unequally, with reduced responses as wavelengths deviate from peak excitation^13,49,50^. Therefore, one reason potentially underlying the reduced search response in 660 nm light is that this wavelength only partially stimulates the zebrafish long wavelength opsins (*opn1lw1* and *opn1lw2*) (Fig. 3A)^3,39,51^. We focused on long wavelength mediated changes as these were highly consistent across assays. Other studies have shown that opsins exhibit an intensity-dependent change in phototransduction, prior to saturation^52–55^. Therefore, we reasoned that increasing photic stimulation by increasing intensity may compensate for the attenuated search performance observed at 660 nm. We tested cold white light and 660 nm at approximately 3 (150 µW/cm^2^, 100 lux cold white) and ninefold (450 µW/cm^2^, 280 lux cold white) increases in illuminance (Fig. 3B,C). Our white light intensity controls resulted in nonlinear changes in motor performance in the light and the dark (Fig. 3D,E,H,I,L,M). At 150 µW/cm^2^ motor outputs nominally increased, yet these changes were reversed at 450 µW/cm^2^ and returned response levels observed at baseline intensities. The reversal at brighter light intensities suggests a saturation in photo-responsiveness. This observation is in line with prior studies that show that very intense light becomes aversive to larval zebrafish^56^. Similarly, intensity of 660 nm light also presented a nonlinear impact on motor performance (Fig. 3F,G,J,K,N,O). Notably, even during enhanced performance (e.g., 150 µW/cm^2^), motor performance did not recover to cold white light levels (t-test difference between cold white light and 660 nm for TTA dark period: 50 µW/cm^2^ *t(333)* = 7.797, *p* < 0.0001; 150 µW/cm^2^ *t(151)* = 3.871, *p* = 0.0002; 450 µW/cm^2^ *t(153)* = 5.637, *p* < 0.0001). Therefore, the right-shifted wavelength, 660 nm, is attenuating search performance in an intensity independent manner.

### Combining wavelengths yields distinct patterns of search behavior

**Figure 4:**
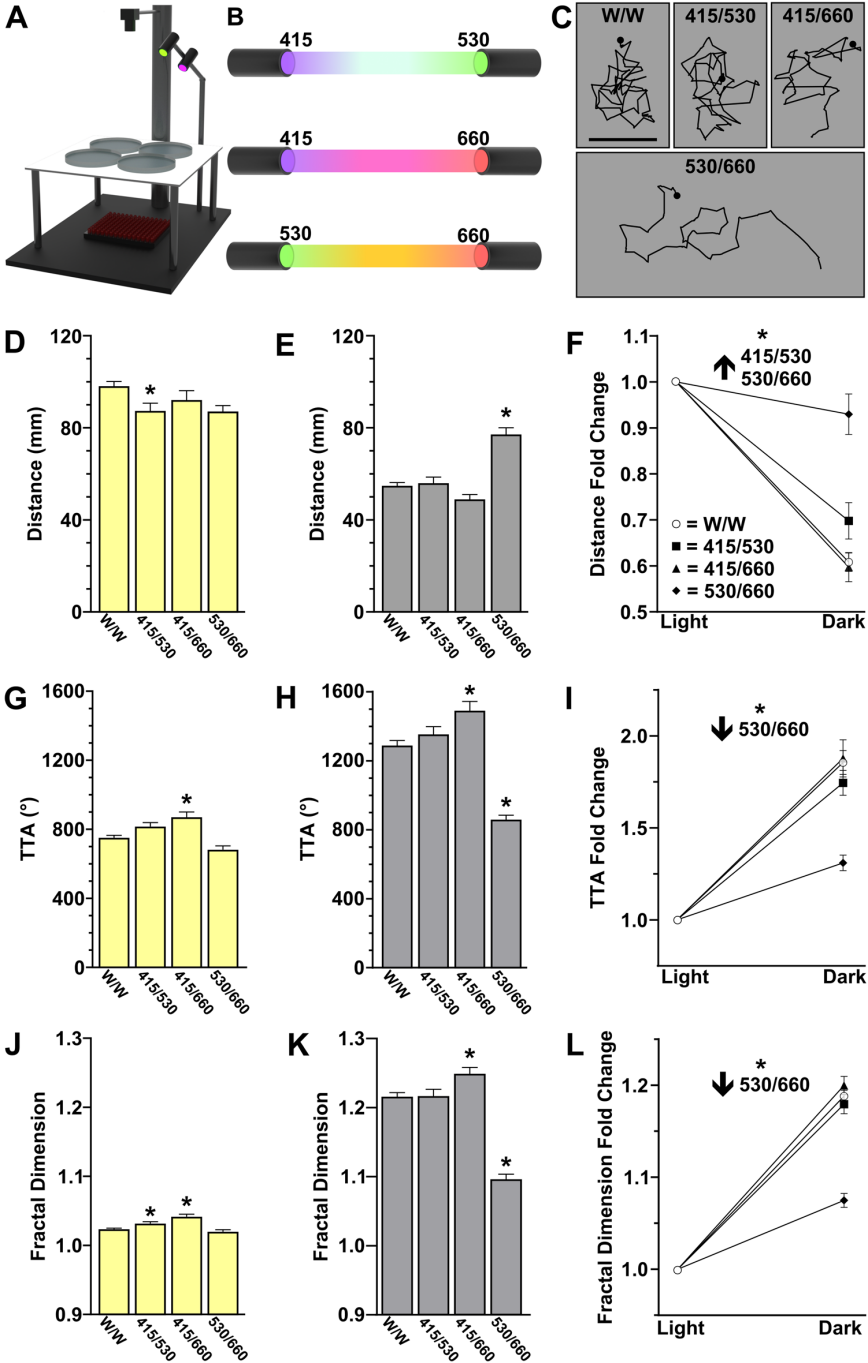
Exposure to two chromatic wavelengths recapitulates white light behavior. (**A**) Behavior rig set up for two LED experiment. (**B**) Visualization of combined wavelengths. (**C**) Representative local search tracks for dual cold white light controls (W/W), combined 415/530 nm, combined 415/660 nm, and combined 530/660 nm light exposure. Scale bar 10 mm. (**D**–**L**) Quantification for the light ON (**D**, **G**, **J**) and OFF (**E**, **H**, **K**) responses and fold change between photoperiods (**F**, **I**, **L**) for each motor metric. Asterisks indicate *p* ≤ 0.05 from white light control. N’s: W/W = 215, 415/530 nm = 94, 415/660 nm = 80, 530/660 nm = 63.

In natural environments, light is not as chromatically narrow as LEDs^5,57^. Therefore, we next wanted to determine how search behavior is impacted by exposure to two wavelengths (Fig. 4A,B). We reasoned that constraining testing conditions to two simultaneous wavelengths would be sufficient to demonstrate potential wavelength-interactive modulation. From within our focused set of three wavelengths, we tested 415/660, 415/530, and 530/660 nm combinations. Like our intensity experiments above (see Fig. 3); we show that increased irradiance from a dual cold white light configuration or dual color light exposure did not disrupt overall search motor output (Fig. 4C). We hypothesized that dual wavelengths would impact search behavior as either (1) driving a response similar to the average of the two wavelengths, or (2) a “winner take all” response where behavioral outputs mirror a single wavelength. Surprisingly, combining 415/660 and 415/530 nm wavelengths resulted in search responses largely consistent with the cold white light control. Only minor, although significant, deviations were observed compared to cold white light (Fig. 4D–L). These data did not support either of our prior predictions. For instance, while 530 nm exposure presented a decrease in total turning and an increase in distance travelled during the local search, the 415/660 nm combination (which averages to 537.5 nm) displayed neither modulation (Fig. 4G,J). Conversely, the combination of 530/660 nm did not restore cold white light motor patterns; instead, outputs were consistent with overall depressed local search across all motor metrics tested (less change in distance, weaker turning, and weaker localized movement). Thus, the local search response differentially incorporates chromatic visual stimuli across the visual spectrum to drive search behavior.

### Outward search performance strength but not transition is affected by wavelength

**Figure 5:**
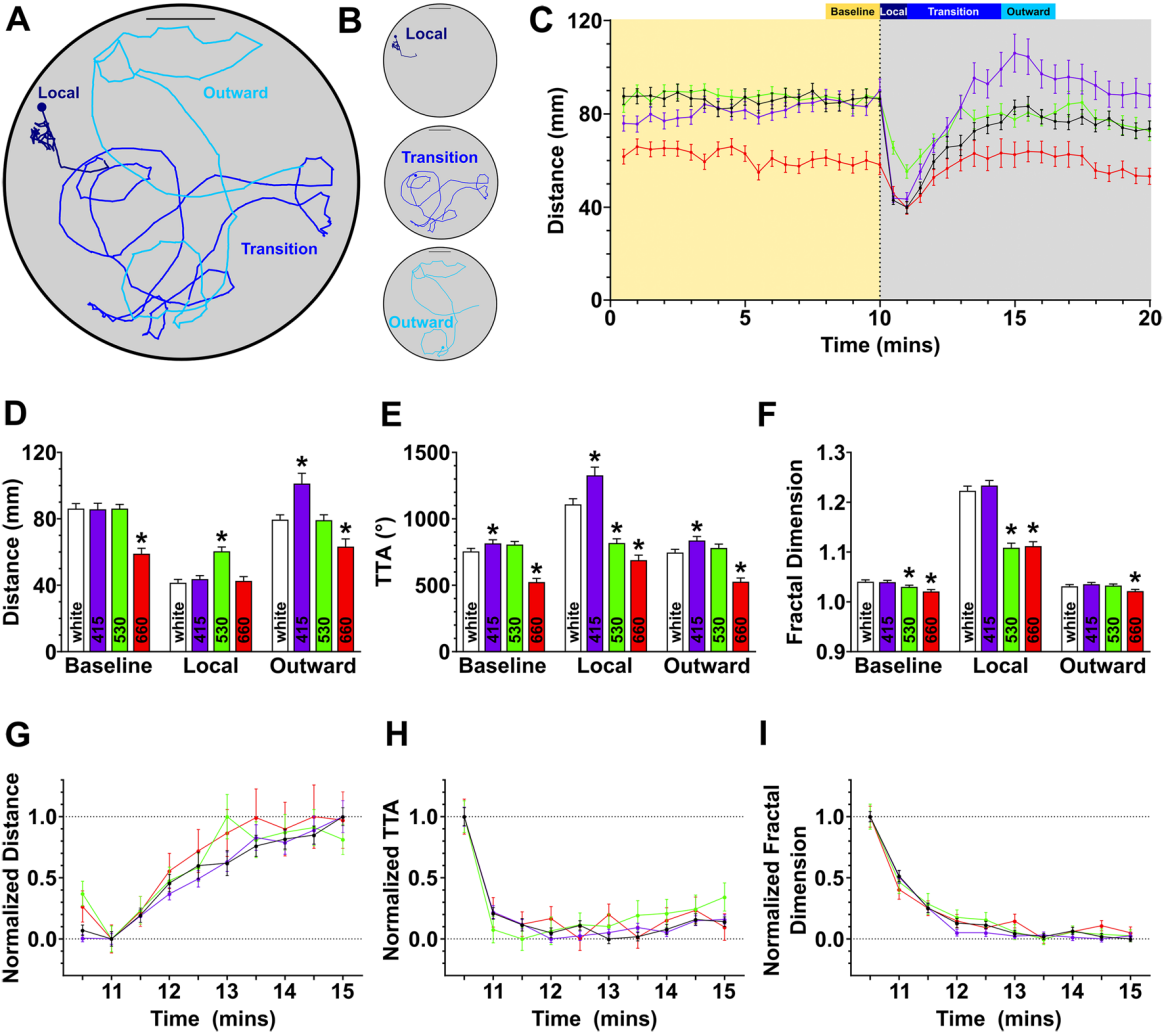
Locomotor modulations persist during outward search behavior. (**A**) Representative track highlighting the local (dark blue line), transitional (blue line), and outward (light blue line) periods. Scale bar 20 mm. (**B**) Isolated subsets of (**A**) showing local, transition, and outward motor paths. Scale bar 20 mm. (**C**) Time-course displaying the change in distance over time. Dotted vertical line at 10 min indicates the loss of illumination. Baseline, local, transition, and outward periods are defined above. (**D**–**F**) Quantification of distance (**D**), TTA (**E**), and fractal dimension (**F**) across baseline, local, and outward periods. Asterisk indicates *p* ≤ 0.05 from white light control within that period. (**G**–**I**) Normalized responses during the transition from local to outward search showing the rate of change during the first five minutes after the loss of light. N’s: cold white light = 74, 415 nm = 73, 530 nm = 80, 660 nm = 67.

A standard feature of search strategies is that local search for nearby resources will transition to an outward exploratory search^25,27^. Indeed, larval zebrafish local search transitions to an outward search strategy, which displays more outward movement and serves as a behavioral mechanism to find distal sources of light (Fig. 5A–C)^14^. Prior work has suggested that the local to outward search transition in zebrafish is at least partially regulated by deep-brain photoreceptors^14^. Therefore, we examined how wavelength impacts outward search and the transition between search states. Zebrafish typically begin their transition from the local to outward search at about a minute after the loss of light, and fully reach outward search by 4 min in a sustained dark environment (Fig. 5A,B)^14^. To characterize changes in search strategy transitions and outward search, we extended our recording timeline to 10 min during baseline illumination and 10 min following light extinction (Fig. 5C). Across wavelengths, this extended recording strategy recapitulated local search behavior changes previously observed (Fig. 5D–F, baseline and local intervals). In addition, outward search behavior was also modulated, revealing the extended impact of wavelength on light search performance (Fig. 5D–F, outward interval). As an example of this modulation, 415 nm light displayed hyperactivity while 660 nm light showed decreased turning and distance while larvae were performing outward search (Fig. 5D–F). These data show that the wavelength-dependent trends during local search extend to outward search motor responses.

Next, we wanted to determine whether wavelength altered the transition latency between local to outward search states. As the various motor metrics displayed changes in magnitude across wavelengths, we standardized data within wavelengths to allow direct comparisons (see Fig. 5C). Despite wavelength-dependent modulation on the magnitude of motor outputs, no prominent changes were observed in the transition rate between search states across any of the tested metrics (Fig. 5G–I). Therefore, the neural signaling driving the transition between search strategies is wavelength independent, yet wavelength broadly influences the strength of motor outputs underlying search pattern behavior.

### Motor performance is likely modulated by non-visual photoreceptors at long wavelengths

**Figure 6:**
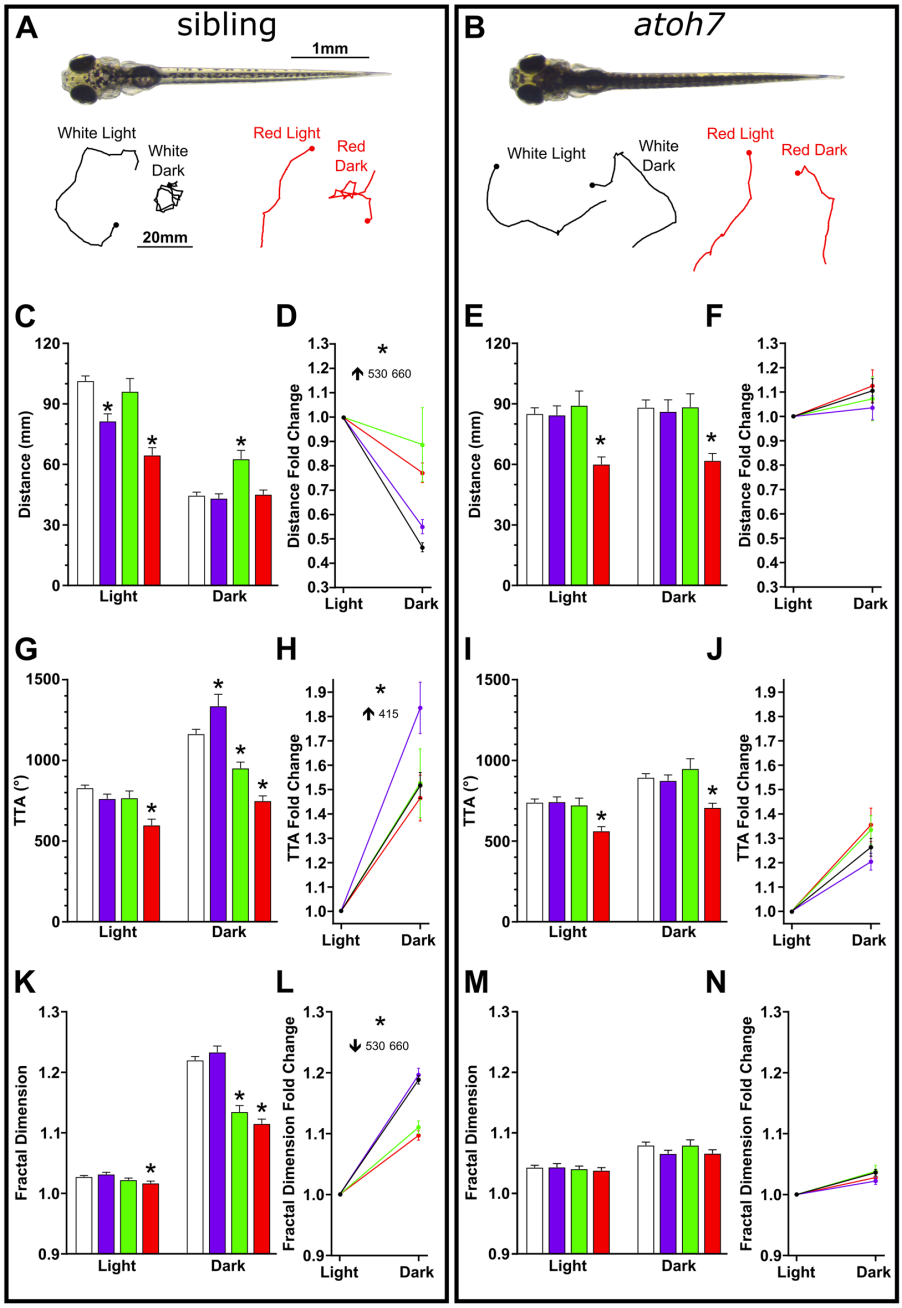
Long wavelengths modulate search behavior in blind larvae. (**A**, **B**) Representative image of 6 dpf sibling (+ /?) and homozygous *atoh7* mutant (–/–). Below are illustrative path trajectories following the loss of light for each tested condition. C-N) Quantification of motor metrics for siblings (left) and mutants (right). Showing distance (**C**–**F**), TTA (**G**–**J**), and fractal dimension (**K**–**N**). Asterisks in bar graphs indicate *p* ≤ 0.05 to white light controls during that period (base or dark). For fold change line graphs asterisk shows *p* ≤ 0.05 to white light control where effected wavelength is indicated, and arrow shows direction of change. N’s: sibling cold white light = 142, 415 nm = 56, 530 nm = 50, 660 nm = 57; *atoh7* white light = 114, 415 nm = 48, 530 nm = 36, 660 nm = 50.

Teleost have a multitude of nonvisual opsins present in their central nervous system, also known as deep brain photoreceptors^10,11,13,14,58–61^. These photoreceptors are involved in functions such as entraining the biological clock and have previously been shown to modulate visuomotor behaviors. To address whether deep brain photoreceptors are involved in the wavelength-dependent modulation of search motor parameters, *atoh7* mutant zebrafish were tested (Fig. 6). These mutants lack retinal ganglion cell (RGC) projections and have a loss of all spontaneous or evoked retinal responses to the brain^62–65^. Blind larvae still modulate their behavior following the loss of light indicating a role for non-visual deep brain photoreceptor based mechanisms^11,14,28^. Our earlier work demonstrated that retinal input was necessary to induce characteristic features of local search (increased local movement), yet dispensable for driving increased turning following the loss of illumination^36^. We performed in-crosses of *atoh7* carriers and tested sighted siblings and blind mutants, which could be phenotypically determined by 4 dpf as loss of vision causes robust melanophore expansion and a dark coloration (Fig. 6A,B). Sighted siblings largely recapitulated earlier results in both magnitude of motor outputs and fold change following light transition (Fig. 6C,D,G,H,K,L). Conversely, in blind larvae, 415 and 530 nm light had minimal impact on visuomotor performance (Fig. 6E,F,I,J,M,N). Surprisingly, long wavelength 660 nm light still attenuated motor performance in blind mutants (Fig. 6E,I). Consistent with prior studies^36^, we still observed a significant increase in turning following the light transition in blind larvae (light = 738.08 ± 21.48, dark = 892.34 ± 24.94, 2-tail paired *t*-test, *t(113)* = *6.478*, *p* < 0.0001) (Fig. 6I, Supplementary Table 2). Therefore, non-visual photoreception was intact and impacted by 660 nm light. These results suggest that deep brain photoreceptors contribute to motor performance in response to long wavelength illumination and a potentially generalized mechanism suppressing motor output at long wavelengths.

## Discussion

Chromatic vision, or color vision, provides key information about the environment. Color can provide information about prey, provide camouflage, warn predators, attract mates, and indicate social status ^66–68^. Moreover, animals have evolved a diversity of light-sensitive proteins, opsins, to specifically respond to distinct chromatic inputs. Moreover, environmental conditions can drive plasticity with visual processing by changing opsin function and expression^69–71^. Despite the importance of chromatic input on how animals respond to the environment, how specific wavelengths within the visual spectrum modulate behavioral performance is incompletely understood. Here we examine how specific wavelengths of light modulate a light-search behavior in larval zebrafish. Search behavior undergoes well-defined transitions, performed with a discreet set of motor patterns, and is conserved in teleost evolution^14,28^, thus providing a tractable behavior to resolve wavelength-specific modulation, which will likely be broadly informative. Like other species, photic and wavelength-dependent information is crucially important to fish, which respond to photic information using retina and non-retinal opsins. Indeed, even in fish with naturally evolved blindness (e.g., cavefish), or deep-sea species, which together represent groups with no or limited light experience, retain functional opsins or display adaptations to capitalize on even limited chromatic inputs^72–74^. Our work shows that narrow spectrum chromatic light can drive acute and distinct changes in etiologically relevant behavior. Yet, light wavelength does not unconditionally influence all motor parameters (e.g., no changes in motor asymmetry or local-to-outward search transition). Thus, our work provides evidence of context dependent modulation by chromatic inputs.

### Experiencing different wavelengths in the environment

Demonstrating wavelength dependent modulation of behavior poses the question of when or if a species may encounter enrichment of a specific chromatic light source. For aquatic species, water naturally enriches for blue and green areas of the visible spectrum^75,76^. However, aquatic environments vary greatly, and increased detritus can red-shift ambient light^77,78^. These transformations to longer wavelengths are associated more often with freshwater environments, where aquatic and amphibious species have mechanisms to enhance long-wavelength sensitivity, indicating these spectral shifts are biologically relevant and animals have evolved mechanisms to take advantage of this enrichment^51,79^. Even in terrestrial environments debris in the atmosphere, such as smoke or dust, favor long-wavelengths that produce a reddish sky—a process known as Rayleigh scattering^80–82^. This process also produces an increase in long, reddish, wavelengths at dusk. Therefore, there are various environmental conditions that selectively enrich long wavelengths. In addition, aquatic environments can also favor specific wavelengths based on increasing water depth, which progressively filters out short to long wavelengths. Although this depth filtering is unlikely to be germane to zebrafish, such spectral filtering is likely important in marine species, which prior work has shown plasticity in opsins based on environmental parameters and preferred habitat in terms of water depth^83,84^. Natural environments, like the native range of zebrafish through India and surrounding countries, experience wide ranges of light intensity, which the fluctuation and intensity are not captured in typical lab or experimental setups. The intensity of light can modulate, up to a saturation point, opsin activity^85,86^. Indeed, very bright white light can reverse the typical phototactic response, negative phototaxis, of larval zebrafish, demonstrating behaviorally relevant intensity dependent change^42^. Here, we test a relatively narrow, compared to the natural environment, range of light intensities, and observe significant yet nominal changes with no consistent pattern. Therefore, intensity is likely important for the perceived valence of a light source with a smaller role on specific patterns of motor performance.

Another consideration is the laboratory environment. Animals are exposed to narrow spectrums of visible light in a wide range of experimental approaches. These experiments include optogenetics and functional imaging where transgenic proteins require relatively intense light of a specific wavelength—typically in the visible range. Here, we show that all wavelengths elicit search pattern behavior, suggesting all visible wavelengths minimally preserve basic visuomotor output. Nonetheless, the observed differences suggest wavelength-dependent modulation in brain function do occur and therefore a potential consideration for in vivo experiments using distinct wavelengths for manipulation and/or recording.

### Wavelength has selective impacts on motor performance

Prior studies have shown that, in larval zebrafish, exposure to narrow spectrum wavelengths in the visible range drive distinct patterns of activity in the brain during light on periods and following light extinction^17,19,87,88^. Wavelength-dependent changes in neural activity are consistent with our findings showing changes in search behavior performance. However, we also establish that not all motor features we quantified were wavelength sensitivity, even those closely aligned with search behavior. For instance, the motor asymmetry feature of zebrafish search behavior is wavelength independent. Our prior work suggests motor asymmetry is dependent on retinal input to the thalamus^89^. Conversely, initiation of local search, when motor asymmetry is observed, is also retina dependent and likely driven by optic tectum output, the primary visual processing center in zebrafish^14,90^. Interestingly, we also show the transition from local to outward search is unaffected by wavelength and occurs over a standard time scale under all conditions tested here. Prior work used knockdown to show that somatostatin and melanopsin (opn4a), a non-visual photoreceptor, are necessary for maintaining the timing of search state transition^14^. Mammalian melanopsin is activated by wavelengths at 480 nm^91,92^, which likely overlaps with the emission spectra of our 415 and 455 nm LEDs. Indeed, work in zebrafish suggests melanopsin is activated by short wavelength (420 nm) light^93^. In addition, zebrafish have five melanopsin genes^94^. These diverse fish melanopsins show high sequence similarity to mammalian melanopsin, yet opn4.1 in a heterologous cell system shows peak absorbance at 403 nm^94^, suggesting some variability in melanopsin sensitivity in zebrafish, although this remains incompletely understood. The additional influence of non-visual melanopsin signaling may contribute to the prominent impact short wavelengths have on search motor performance as seen in the motor spectra analysis (Fig. 1M). As we observed changes in the magnitude of search motor outputs, yet not transition latency, suggests potentially independent neural actions driven by photo-mediated signaling and general G-protein coupled receptor signaling^95–98^. Supporting this idea, the loss of non-visual opsins valopa/b or tmt2 disrupt early development and neuromodulatory functions, respectively, in a seemingly light independent manner, implicating deep-brain photoreceptors may exert physiological roles outside of active photoreception^99,100^. Altogether, our work complements prior studies showing chromatic inputs differentially drive activity in the brain by establishing new and distinct behavioral changes instructed by wavelengths across the visual spectrum.

### Spectral tuning of search behavior

We observed a general trend in wavelength-dependent modulation of search performance, where short to long wavelengths drove increased and decreased changes in search strength, respectively. This overall trend may correspond with other biological attributes associated with specific wavelengths. For instance, in humans, an increasing area of interest is how blue wavelengths emitted by digital devices impact circadian cycles and sleep^100,101^, which is recapitulated in animal models as well^102^. Conversely, zebrafish raised in short wavelengths leads to increased hatching rates, whereas long wavelengths increased mortality^103^. These studies show that discreet wavelength exposure can impact physiology and behavior across species in diverse ways. Interestingly, a growing body of work suggests that short wavelengths are associated with increased alertness^104–106^. In zebrafish, short wavelengths produce more robust activity in the habenula, an important limbic brain region^107–109^. Therefore, enhances in motor performance at short wavelengths, consistent with our results, may be driven by imposing a state of heightened arousal. Conversely, longer wavelengths still activate responses in the habenula, yet much weaker^107,110^. The attenuated activation in this key limbic reason may explain the reduction of search strength we observed.

A putative caveat with wavelength specific behavioral modulation is that we observed that combining most wavelengths, even those with opposing effects (e.g., 415 and 660 nm) restored behavioral responses to those observed with white light. The only exception was the combination of 530 and 660 nm. These data demonstrate a more complex relationship between chromatic signaling and behavior modulation. However, from our combined wavelength results we did notice a correlation with our PCA analysis and clustering (Fig. 1). In the PCA, individual wavelengths were generally represented as sub-regions of the overall cold white light. Surprisingly, it appears that combining wavelengths from different sub-regions, 415 nm and 660 nm for example, restored white light behavioral responsiveness. Conversely, combining wavelengths from largely overlapping regions in the PCA, 530 and 660 nm, did not recapitulate white light motor patterns. This data suggests that wavelengths across the visual spectrum potentially cluster into categories with different functional relevance to behavior. The underlying basis of this clustering may be the asymmetric distribution of chromatic responsiveness across the zebrafish retina or potential spectral opponency interactions between wavelengths^88,111,112^. Therefore, typical search patterns may only be evoked once signals are received by appropriate zones from across the retina or opponency groups are stimulated. In addition, stimulation of distinct opsins or combinations of opsins may elicit unique pathways. Many of the wavelengths tested stimulate, at least partially, several zebrafish opsins (see Table 1). As an example, during outward search (Fig. 5), 415 nm light caused overcompensation in distance that was not observed 530 nm, providing some evidence that the subsets and magnitude of opsins stimulated could be triggering distinct functional pathways. In the motor spectra analysis, there was a relatively equal impact of each wavelength in the visual spectra on search motor response change (Fig. 1M). However, we note that during the transition from short to longer wavelengths there is progressive loss of short-wavelength opsin contributions (see Table 1). Thus, one hypothesis for the changing patterns of behavior is the differential mix and proportions of opsin signaling stimulated by any specific wavelength. Although the current study does not aim to undercover the mechanism, our results suggest that the full set of complementary and/or antagonistic interactions between opsin signaling pathways triggered by more etiological white light interact to produce typical behavioral patterns observed. Overall, these results demonstrate behaviorally relevant patterns in chromatic responses that are discernable by analyzing search behavior performance and therefore may be a useful tool for future studies to understand chromatic interactions on neurophysiology and behavior, which has consequences for understanding animal behavior in natural and laboratory environments.

## Methods

### Zebrafish husbandry and rearing

The West Virginia University Institutional Animal Care and Use Committee approved all experiments, which were designed to follow all relevant guidelines, regulations, and ARRIVE guideline standards. Zebrafish of the wildtype strain Tübingen long fin (TL) were used in all experiments. This includes the zebrafish with a null mutation in the *atonal bHLH transcription factor 7* (*atoh7*^*sa16352*^), which we maintained in a TL background. Mutants and siblings were sorted phenotypically, as mutants have expanded melanophores and are visibly darker. Embryos and larvae were raised in 10cm Petri dishes with approximately 30 mL of E3 embryo medium (5 mM NaCl, 0.17 mM KCl, 0.33 mM CaCl2, 0.33 mM MgSO4, and 1 mM HEPES, pH 7.3). These embryos were raised in an incubator set to 28.0 °C with a 14:10 light:dark cycle. Light intensity in the incubator was 60 µW/cm^2^. Dishes were cleaned of any dead or deformed embryos and renewed with fresh E3 daily. Zebrafish embryos were raised until they were 6 to 7 days post fertilization (dpf), at which they were used for behavior testing.

### Behavior testing

Recordings were captured on a custom behavior recording rig^35^. The rig included an infrared illuminator (940 nm, CMVision Supplies) and captured images using an µEye IDS1545LMM CMOS camera (1st Vision) with a 12 mm lens (Thorlabs) (Cat# MVL12WA), and an infrared filter (780 nm long pass) (Cat# FGL780). Visual illumination was provided by mounted LEDs (Thorlabs). Cold white light (Cat# MCWHL5, 6500 K) and infrared (Cat# M970L4) were used as the positive and negative controls, respectively. Wavelengths used for the experimental groups included 415 nm (Cat# M415L4), 455 nm (Cat# M455L4), 490 nm (Cat# M490L4), 530 nm (Cat# M530L4), 565 nm (Cat# M565L3), 595 nm (Cat# M595L4), 660 nm (Cat# M660L4), and 730 nm (Cat# M730L5). Warm white light (Cat# MWWHL4, 3000 K) was used for the initial experiment. Absorbance spectra for the four retinal cone opsins and relative absorbance at different wavelengths were generated using published formulas and absorbance peaks^33,39,51,87^. Light intensity from each LED was adjusted to an irradiance of 50 µW/cm^2^ at the center of the stage. As the cold and warm white light sources were composed of a broad and uneven mix of wavelengths, we also measured illuminance, which for cold and warm white light LEDs was 30 and 28 lux, respectively. Irradiance (µW/cm^2^) was measured using ILT2400 optical meter (International Light Technologies) and white light source illuminance (lux) with a SDL400 light meter (Extech). The only exception is the intensity experiment, which also had groups set at 150 and 450 µW/cm^2^. Two different LEDs were used during the alternating light experiment and the two simultaneous light experiments. In both cases, each individual LED was set to 50 µW/cm^2^ and the controls were done using two cold white light LEDs. Custom IDL based DAQtimer software was used to control illumination, recording timing, and tracking parameters consistent with prior studies. For experiments with repeated trials the inter-trial interval was 150 s of light ON conditions with intensity matched to the recording conditions. The camera was set to record the zebrafishes’ position at a frame rate of 10 fps.

### Behavior analysis

Custom DAQtimer software converted the recordings into positional data for each larva captured at 10 Hz^14,29^. This positional data was used to calculate four measures: distance, fractal dimension, total turn angle (TTA), and match index (MI). Any larvae that did not have 100 positional points were excluded from the analysis. N’s shown in all figure legends were used to calculate all motor metrics and related measures unless otherwise stated. Distance was defined as the length of the track during a recording period. Fractal dimension is an index of the complexity of a geometric pattern and was used to gauge the tortuosity of the path trajectory and calculated using a box counting method^113^. TTA is the total degrees of angular displacement regardless of direction. Distance, TTA, and fractal dimension are measured over 30 s intervals, which measures from these intervals are averaged for analysis. The MI is a binarized value based on net turn angle, a measure that is the sum of angular displacement over 30 s where leftward and rightward movement is represented as negative and positive degrees displacement, respectively. MI measures whether the direction of turning over successive trials from an individual matched the direction of the first dark period. Fold change was calculated dividing every paired light to dark recording to the baseline (light ON) response, which standardized baseline responses to 1. For calculating fold change from MI all values were transformed by adding 1. This transformation binned values between 1 and 2 to avoid dividing by 0. The average light or dark MI for a sample is the ratio of trials that preferential turn in the same direction as the first dark period. Larvae that had weak directional turning (between − 100° and 100°) with limited predictive value during the first dark trial were excluded from MI analysis. Typically, only 2–3 larvae per dataset were excluded based on this criterion. The MI excludes the first light or the dark are averaged to avoid bias from the self-matching 1st dark period. All other metrics were averaged across the four light periods and the four dark periods, and then each metric was averaged across all samples for light and dark.

### Principal component analysis

Principal component analysis (PCA) was performed in R using the “prcomp” function part of the base R stats package. To reduce overrepresentation of cold white light individuals we took a random subset of 70 individuals to be used in the analysis gathered with the “sample_n” function. PCA was performed on normalized fold change data for fractal dimension, distance, and total turn angle for all wavelengths. Eigenvalues and variance were calculated using the package “FactoMineR”. Individual datapoints were grouped together by creating ellipses which encompassed 90% of data points for each wavelength.

### Phototaxis assay

The behavior apparatus was adjusted to measure the phototaxis of zebrafish to different wavelengths of light. The white, 415 nm, 530 nm, and 660 nm were angled to project light from underneath the behavior stage without blocking the infrared light necessary for tracking. The multiplex setup was replaced with on 12 cm square petri dish. The LED light was projected at the center of the dish to create a light spot with an approximate intensity of 100 µW/cm^2^ and a radius of about 1.25 cm. Apertures were made from cardstock paper by punching a hole with a diameter of approximately 1 cm and controlled the illumination area of the LED. One larva was added to the dish per experiment. The light series included an initial 3 min of baseline light acclimation to the overhead cold white light, 2 min of recording in the cold white light, followed by a final 5 min of recording with just the light spot as illumination. Recording periods were broken down into 30 s increments for analysis. Photos of the light spot relative to the dish were used to generate ROIs matching light spot diameter for analysis. The light spot radius was adjusted to approximately 2.5 cm for all trials. Data was calculated as percentage of the track inside the light spot as represented by a digital ROI in the same DAQtimer software used above.

### Outward search

Larvae were exposed to an altered light series while recorded in the multiplex setup. Following a 5-min acclimation period, larvae were recorded for 10 min in 50 µW/cm^2^ of either cold white, 415 nm, 530 nm, or 660 nm light. Recording continued after the loss of light for another 10 min. Metrics were binned into 30 s increments. The last 2 min of the light, the first minute of the dark, and 2.5 min in the dark following 4.5 min of darkness were averaged and statistically compared to assess the behavior during baseline, the local search, and the outward search respectively. For each metric, 30 s intervals from the local search to the beginning of the outward search were normalized and statistically compared to determine if the rate of change and/or timing of these behaviors differed between the wavelengths. For quantifying local to outward rate of transition a sustained difference for a least two consecutive timepoints was required to be deemed significant to reduce the impact of scaling and any potential acute motor fluctuations.

### Statistical analysis

Comparisons were made across wavelengths within baseline and dark conditions using One Way Analysis of Variance (ANOVA) followed by the Dunnett correction for multiple comparisons when all statistical comparisons were to white light. For the motor spectra, alternating light experiment, and the light intensity experiment, where multiple comparisons were made within entire datasets, ANOVA was run and Tukey correction performed. Paired data (such as comparisons between light and dark periods from the same larva) were made using paired t-tests, while unpaired comparisons were done using the Student’s t-test. Fold change data was compared to the hypothetical value of 1 indicative of no change using One sample t-tests. Normality was determined using the Shapiro–Wilk test. For non-normal data, such as the MI, non-parametric tests were used. For the MI, data was compared to 0.5 (random), using the Wilcoxon rank-sum test. Group comparisons were performed using the Kruskal Wallis test followed by Dunn correction for multiple comparisons. Motor spectra was calculated using fold change data for distance, fractal dimension, and TTA that was rescaled to a 0 to 1 scale through min–max normalization. The rescaling for distance was modified by subtracting each sample value from the maximum so that wavelengths that evoked the greatest change would be matched to 1 and allow more direct comparison between other motor metrics. Principal component analysis (PCA) was performed in R using the “prcomp” function in the base R stats package. To reduce overrepresentation of cold white light individuals we took a random subset of 70 individuals using the “sample_n” function. Eigenvalues and variance were calculated using the package “FactoMineR”. Individual data points were grouped together by creating ellipses which encompassed 90% of data points per wavelength. All comparisons with a *p* ≤ 0.05 were considered statistically significant. All statistics were performed in Graphpad Prism and were two-tailed. All error bars or line envelopes show standard error of the mean (SEM).

### Supplementary Information

Supplementary Information.

## Data Availability

Data is available at Mendeley Data 10.17632/p2swf4v5hp.1. Data and reagents are also available upon request.
