# Supplementary material for: Light wavelength modulates search behavior performance in zebrafish
Source: Sci Rep. 2024 Jul 17;14:16533. doi: 10.1038/s41598-024-67262-9 (PMC11255219; doi:10.1038/s41598-024-67262-9)
Supplement: Supplementary file 1 — Supplementary Information. [file 41598_2024_67262_MOESM1_ESM.docx]

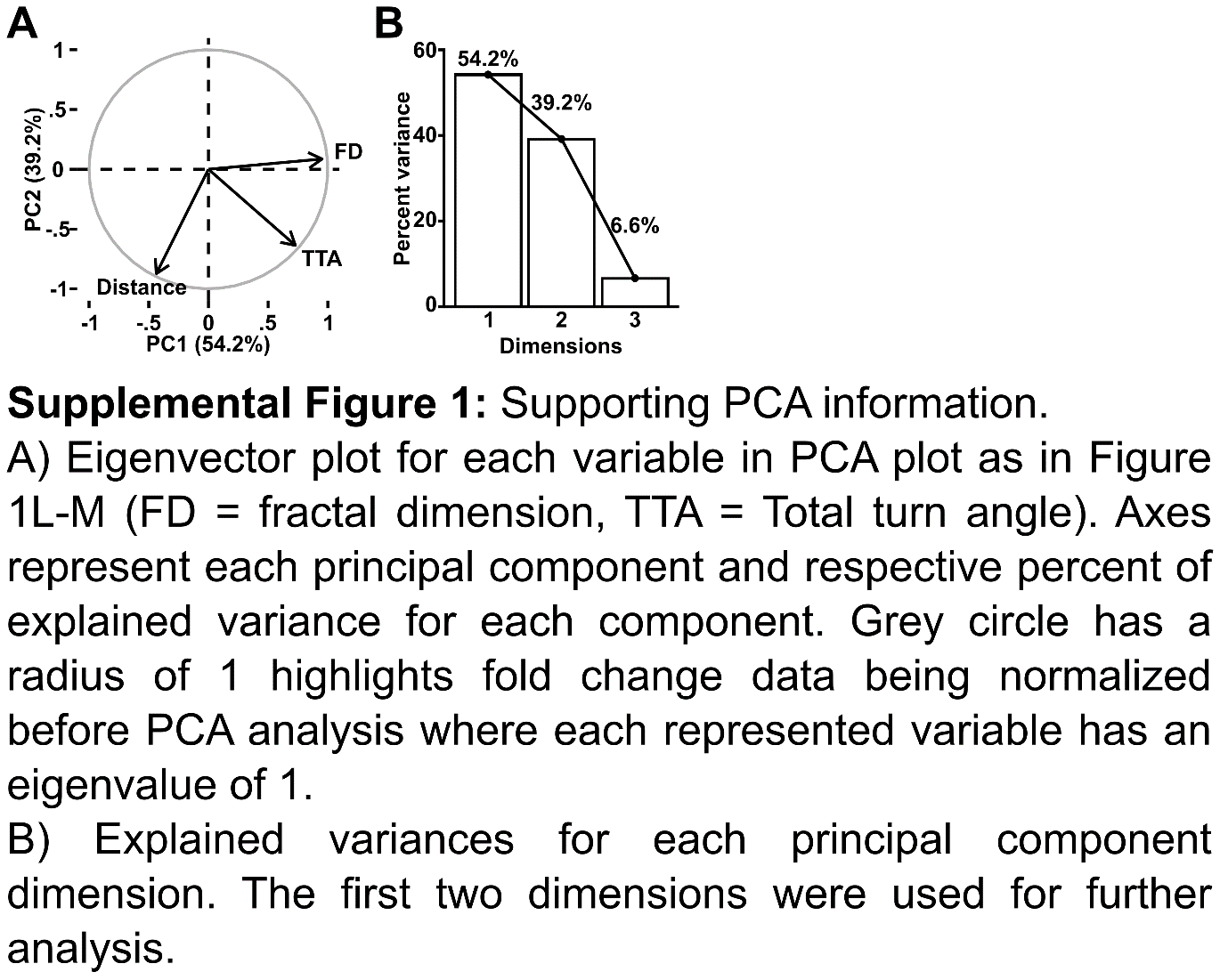


**Supplementary Figure 1**: Supporting PCA information.

A) Eigenvector plot for each variable in PCA plot as in Figure 1L-M (FD = fractal dimension, TTA = Total turn angle). Axes represent each principal component and respective percent of explained variance for each component. Grey circle has a radius of 1 highlights fold change data being normalized before PCA analysis where each represented variable has an eigenvalue of 1.

B) Explained variances for each principal component dimension. The first two dimensions were used for further analysis.


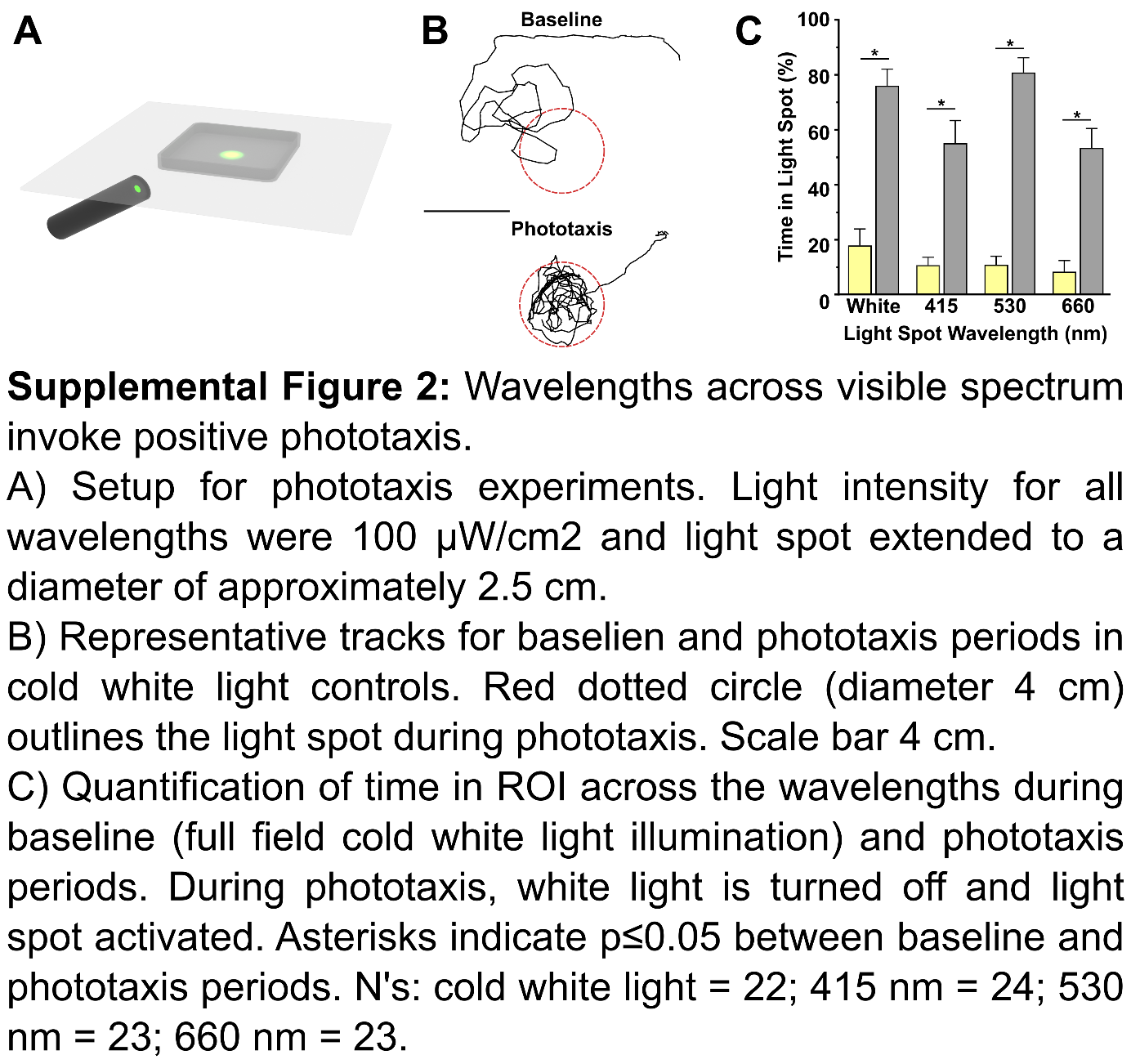


**Supplementary Figure 2**: Wavelengths across visible spectrum invoke positive phototaxis.

A) Setup for phototaxis experiments. Light intensity for all wavelengths were 100 μW/cm^2^ and light spot extended to a diameter of approximately 2.5 cm.

B) Representative tracks for baseline and phototaxis periods in cold white light controls. Red dotted circle (diameter 4 cm) outlines the light spot during phototaxis. Scale bar 4 cm.

C) Quantification of time in ROI across the wavelengths during baseline (full field cold white light illumination) and phototaxis periods. During phototaxis, white light is turned off and light spot activated. Across all tested wavelengths the relative strength of phototaxis was significantly variable (Kruskal-Wallis *H*(3)=12.39, *p*=0.0062), yet no significant differences were observed when compared to white light (white vs. 415 nm *p* = 0.1803; 530 nm *p* > 0.9999; 660 nm *p* = 0.0514). Asterisks indicate p ≤ 0.05 between baseline and phototaxis periods. N's: cold white light = 22; 415 nm = 24; 530 nm = 23; 660 nm = 23.


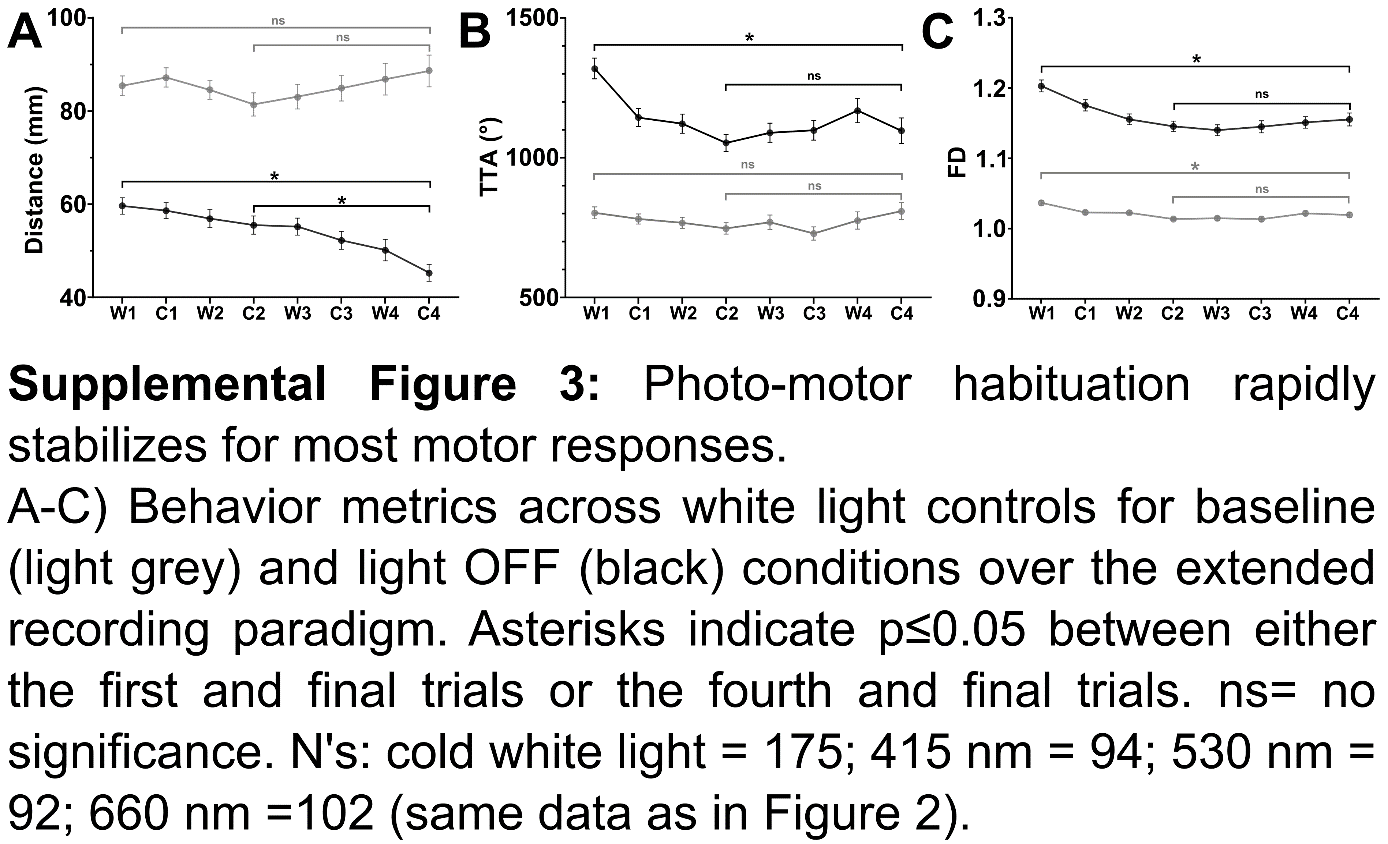


**Supplementary Figure 3**: Photo-motor habituation rapidly stabilizes for most motor responses.

A-C) Behavior metrics across white light controls for baseline (light grey) and light OFF (black) conditions over the extended recording paradigm. Asterisks indicate p ≤ 0.05 between either the first and final trials or the fourth and final trials. ns = no significance. N's: cold white light = 175; 415 nm = 94; 530 nm = 92; 660 nm = 102 (same data as in Figure 2).


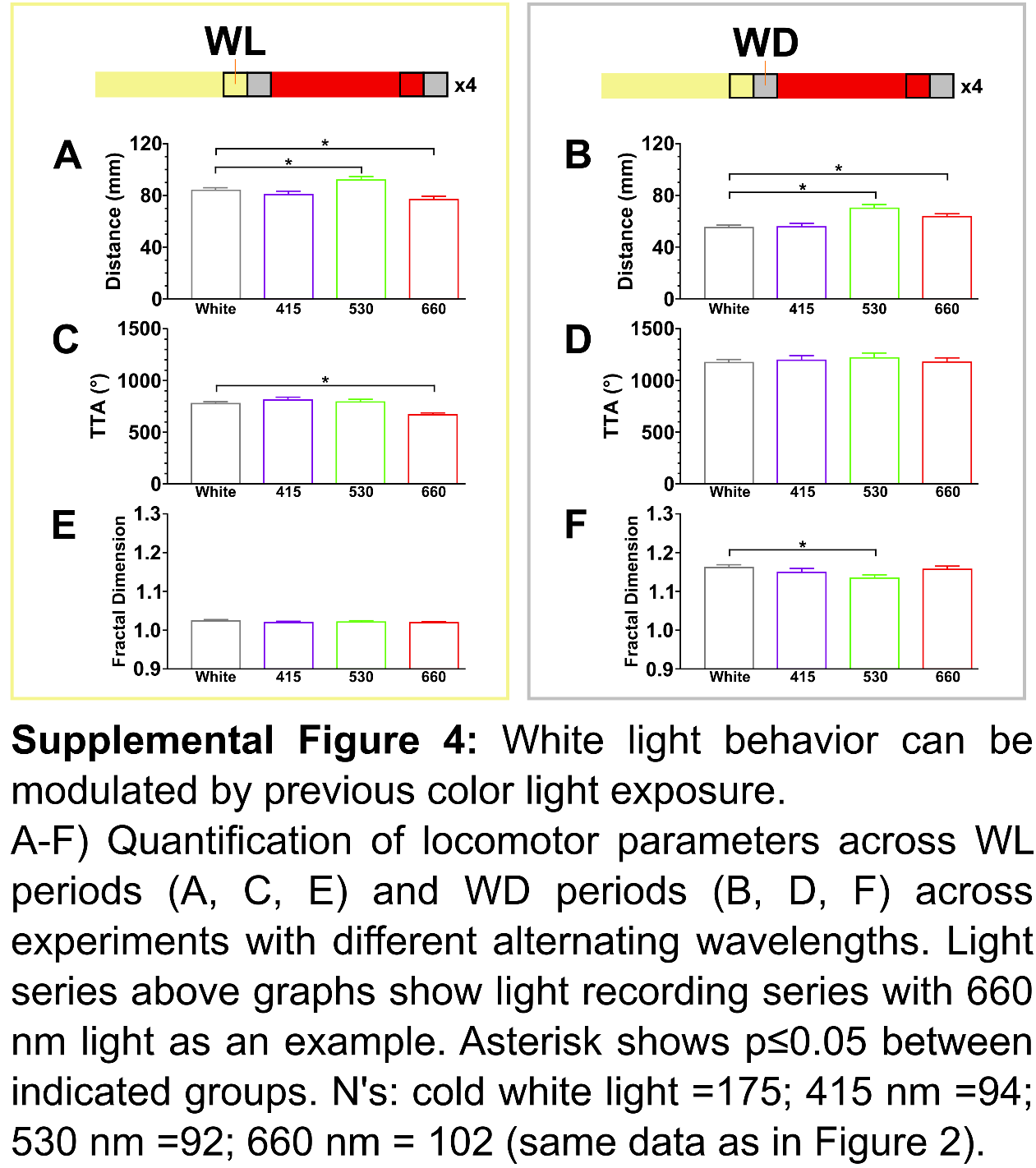


**Supplementary Figure 4**: White light behavior can be modulated by previous color light exposure.

A-F) Quantification of locomotor parameters across WL periods (A, C, E) and WD periods (B, D, F) across experiments with different alternating wavelengths. Light series above graphs show light recording series with 660 nm light as an example. Asterisk shows p ≤ 0.05 between indicated groups. N's: cold white light = 175; 415 nm = 94; 530 nm = 92; 660 nm = 102 (same data as in Figure 2).

**Supplementary Table 1**: One sample t-test comparing light to dark fold change data.

| **Metric** | **Wavelength** | **Mean ± SEM** | **N** | ***p* value** | **t statistic** |
| --- | --- | --- | --- | --- | --- |
| Distance | Cold White | 0.686 ± 0.024 | 564 | **<0.0001** | 13.22 |
|  | Warm White | 0.679 ± 0.048 | 59 | **<0.0001** | 6.63 |
|  | 415nm | 0.668 ± 0.040 | 64 | **<0.0001** | 8.24 |
|  | 455nm | 0.653 ± 0.040 | 64 | **<0.0001** | 8.65 |
|  | 490nm | 0.745 ± 0.040 | 68 | **<0.0001** | 6.43 |
|  | 530nm | 0.857 ± 0.049 | 62 | **0.0045** | 2.95 |
|  | 565nm | 0.859 ± 0.045 | 77 | **0.0026** | 3.12 |
|  | 595nm | 0.945 ± 0.074 | 59 | 0.4568 | 0.75 |
|  | 660nm | 0.867 ± 0.060 | 59 | **0.0316** | 2.20 |
|  | 730nm | 0.998 ± 0.060 | 54 | 0.9747 | 0.03 |
|  | 970nm | 0.994 ± 0.019 | 65 | 0.7638 | 0.30 |
| TTA | Cold White | 1.724 ± 0.036 | 564 | **<0.0001** | 20.22 |
|  | Warm White | 1.460 ± 0.060 | 59 | **<0.0001** | 7.72 |
|  | 415nm | 2.116 ± 0.102 | 64 | **<0.0001** | 10.89 |
|  | 455nm | 2.119 ± 0.062 | 64 | **<0.0001** | 17.97 |
|  | 490nm | 1.690 ± 0.077 | 68 | **<0.0001** | 8.93 |
|  | 530nm | 1.200 ± 0.052 | 62 | **0.0003** | 3.87 |
|  | 565nm | 1.450 ± 0.058 | 77 | **<0.0001** | 7.78 |
|  | 595nm | 1.428 ± 0.079 | 59 | **<0.0001** | 5.43 |
|  | 660nm | 1.616 ± 0.098 | 59 | **<0.0001** | 6.31 |
|  | 730nm | 1.499 ± 0.075 | 54 | **<0.0001** | 6.63 |
|  | 970nm | 1.041 ± 0.030 | 65 | 0.1793 | 1.36 |
| Fractal Dimension | Cold White | 1.170 ± 0.005 | 564 | **<0.0001** | 36.23 |
|  | Warm White | 1.140 ± 0.011 | 59 | **<0.0001** | 12.57 |
|  | 415nm | 1.227 ±0.009 | 64 | **<0.0001** | 26.12 |
|  | 455nm | 1.219 ± 0.011 | 64 | **<0.0001** | 19.74 |
|  | 490nm | 1.164 ± 0.013 | 68 | **<0.0001** | 12.41 |
|  | 530nm | 1.081 ± 0.007 | 62 | **<0.0001** | 10.97 |
|  | 565nm | 1.096 ± 0.009 | 77 | **<0.0001** | 10.32 |
|  | 595nm | 1.082 ± 0.009 | 59 | **<0.0001** | 9.11 |
|  | 660nm | 1.098 ± 0.008 | 59 | **<0.0001** | 12.27 |
|  | 730nm | 1.014 ± 0.010 | 54 | 0.1599 | 1.43 |
|  | 970nm | 1.000 ± 0.004 | 65 | 0.9513 | 0.06 |
| Match Index | Cold White | 1.175 ± 0.016 | 564 | **<0.0001** | 11.00 |
|  | Warm White | 1.133 ± 0.046 | 59 | **0.0059** | 2.86 |
|  | 415nm | 1.160 ± 0.049 | 64 | **0.0019** | 3.24 |
|  | 455nm | 1.197 ± 0.049 | 64 | **0.0001** | 4.04 |
|  | 490nm | 1.174 ± 0.038 | 68 | **<0.0001** | 4.57 |
|  | 530nm | 1.120 ± 0.041 | 62 | **0.005** | 2.91 |
|  | 565nm | 1.198 ± 0.039 | 77 | **<0.0001** | 5.11 |
|  | 595nm | 1.086 ± 0.037 | 59 | **0.0231** | 2.33 |
|  | 660nm | 1.210 ± 0.048 | 59 | **<0.0001** | 4.41 |
|  | 730nm | 1.114 ± 0.033 | 54 | **0.0012** | 3.43 |
|  | 970nm | 1.048 ± 0.029 | 65 | 0.0946 | 1.70 |

**Supplementary Table 2**: One sample t-test comparing light to dark fold change for the siblings and *atoh* mutants across wavelengths.

| **Metric** | **Phenotype** | **Wavelength** | **Mean ± SEM** | **N** | ***p* value** | **t statistic** |
| --- | --- | --- | --- | --- | --- | --- |
| Distance | Sibling | Cold White | 0.466 ± 0.019 | 142 | **<0.0001** | 28.63 |
|  |  | 415nm | 0.551 ± 0.029 | 56 | **<0.0001** | 15.44 |
|  |  | 530nm | 0.887 ± 0.152 | 50 | 0.4598 | 0.75 |
|  |  | 660nm | 0.772 ± 0.040 | 57 | **<0.0001** | 5.68 |
|  | *atoh* | Cold White | 1.105 ± 0.050 | 114 | **0.0381** | 2.10 |
|  |  | 415nm | 1.035 ± 0.049 | 48 | 0.4777 | 0.72 |
|  |  | 530nm | 1.072 ± 0.090 | 36 | 0.4300 | 0.80 |
|  |  | 660nm | 1.125 ± 0.066 | 50 | 0.0640 | 1.90 |
| TTA | Sibling | Cold White | 1.517 ± 0.053 | 142 | **<0.0001** | 9.66 |
|  |  | 415nm | 1.835 ± 0.105 | 56 | **<0.0001** | 7.92 |
|  |  | 530nm | 1.525 ± 0.141 | 50 | **0.0005** | 3.72 |
|  |  | 660nm | 1.465 ± 0.094 | 57 | **<0.0001** | 4.96 |
|  | *atoh* | Cold White | 1.263 ± 0.036 | 114 | **<0.0001** | 7.22 |
|  |  | 415nm | 1.204 ± 0.035 | 48 | **<0.0001** | 5.89 |
|  |  | 530nm | 1.335 ± 0.058 | 36 | **<0.0001** | 5.74 |
|  |  | 660nm | 1.358 ± 0.071 | 50 | **<0.0001** | 5.07 |
| Fractal Dimension | Sibling | Cold White | 1.189 ± 0.007 | 142 | **<0.0001** | 27.17 |
|  |  | 415nm | 1.196 ± 0.011 | 56 | **<0.0001** | 17.74 |
|  |  | 530nm | 1.110 ± 0.011 | 50 | **<0.0001** | 10.53 |
|  |  | 660nm | 1.097 ± 0.008 | 57 | **<0.0001** | 12.44 |
|  | *atoh* | Cold White | 1.036 ± 0.005 | 114 | **<0.0001** | 6.57 |
|  |  | 415nm | 1.022 ± 0.006 | 48 | **0.0003** | 3.92 |
|  |  | 530nm | 1.038 ± 0.010 | 36 | **0.0004** | 3.90 |
|  |  | 660nm | 1.029 ± 0.007 | 50 | **0.0003** | 3.88 |
